# Regional Differences in Prescribing Patterns of Metamizole in Germany Based on Data from 70 Million Persons

**DOI:** 10.3390/ijerph17113892

**Published:** 2020-05-30

**Authors:** Falk Hoffmann, Carsten Bantel, Frederik Tilmann von Rosen, Kathrin Jobski

**Affiliations:** 1 Department of Health Services Research, Carl von Ossietzky University Oldenburg, 26129 Oldenburg, Germany; falk.hoffmann@uni-oldenburg.de (F.H.); ftvonrosen@gmail.com (F.T.v.R.); 2 University Department of Anesthesiology, Critical Care, Emergency and Pain Medicine, Klinikum Oldenburg, 26133 Oldenburg, Germany; carsten.bantel@uni-oldenburg.de

**Keywords:** metamizole, dipyrone, prescribing patterns, prevalence, regional variations, Germany

## Abstract

The non-opioid analgesic metamizole (dipyrone) is commonly used in Germany despite its narrow indications and market withdrawal from several countries. In this study we analyzed prescribing patterns of metamizole focusing on regional differences. The source of data was the “Information system for health care data” which includes data from the statutory health insurance funds for about 70 million Germans. We received aggregated data of individuals with at least one metamizole prescription in 2010 as well as the number of prescribed packages by age, sex, state and district along with the number of insured persons in each stratum. We calculated prescription prevalence stratified by age, sex, state and district. Among 68.4 million insured persons (mean age: 43.6 years; 53.0% female) 5.5 million received at least one metamizole prescription (8.1%, overall 12.2 million packages). Prevalence increased with age, and women received metamizole more often than men. In adults (total prevalence: 9.4%), levels varied between 7.0% (Saxony) and 11.1% (Schleswig-Holstein), whereas on a district level use ranged from 4.3% to 14.3%. In 2010, one of 12 individuals received metamizole at least once. Noticeable were the large regional variations which certainly cannot be explained by patient-related factors.

## 1. Introduction

Metamizole is a non-opioid analgesic. It has been available for nearly a century, and can be placed in the first step on the World Health Organization (WHO) analgesic ladder. It shows good analgesic, antipyretic and spasmolytic efficacy, although studies have been largely confined to the acute setting [1,2,3]. Compared to nonsteroidal anti-inflammatory drugs (NSAIDs) or opioids, metamizole seems to display a favorable profile of renal and gastrointestinal risks [3,4]. Yet, the safety of metamizole has been the topic of a lengthy and controversial debate due to the possibility of agranulocytosis as a grave and potentially fatal adverse effect [3,5,6,7]. Although the true incidence of metamizole-induced agranulocytosis is unclear and varies widely between studies [8,9,10,11,12], many countries including the UK, France, Norway, Sweden, the USA, Canada and Australia have decided to withhold or withdraw market authorization, while it is available over-the-counter (OTC) in others [5]. Germany subjected metamizole to prescription-only status in 1987, and actively narrowed its indications [13].

**Figure 1:**
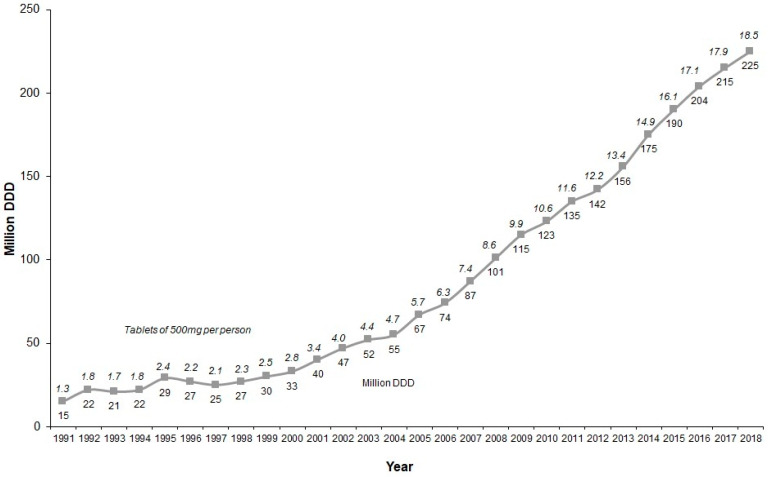
Prescriptions of metamizole in Germany from 1991 to 2018 (according to the Annual Drug Prescription Reports [17,18,19]), displayed as defined daily doses (DDD); 1 DDD = 3 g (lower caption) as well as the calculated number of 500 mg tablets per insured person (upper italic caption).

Nonetheless, metamizole remains a widely used medication in Germany, both in inpatient and outpatient settings [14,15,16]. According to the annual German Drug Prescription Report, prescription volume among outpatients increased fifteen-fold between 1991 and 2018 (Figure 1), from 15 to 225 million defined daily doses (DDD) [17,18,19]. The DDD describes the assumed average maintenance dose per day for a drug used for its main indication in adults [20]. For metamizole, the DDD is 3 g. Correspondingly, a total of 675 tons of metamizole were prescribed to members of statutory health funds in Germany in 2018, which would translate to 18.5 tablets of 500 mg for each insured individual (Figure 1). However, the yearly prescription volume by itself lacks the information necessary to evaluate the true number of patients prescribed metamizole, the doses prescribed, and to assess regional differences. An earlier study using data from one health insurance fund pointed towards a higher prescription prevalence in northwestern Germany, lower levels in southern Germany, and the lowest level in eastern Germany. Prevalence varied visible between states (Bundesländer), with between 4.9% and 8.3% receiving at least one prescription of metamizole in 2009 [15]. To our knowledge, no study has been performed with greater geographic resolution than at the state level, although it can be assumed that marked differences exist even within states. 

The aim of this study is to provide an insight into the number of metamizole prescriptions in outpatient care, with a particular focus on regional differences on the district (Landkreis) level.

## 2. Materials and Methods 

We obtained data from the “Information System for Health Care Data” located at the German Institute of Medical Documentation and Information (DIMDI) [21]. It is based on data from some 70 million individuals covered by all statutory health insurance funds in Germany which are supplied to the Federal Office of Social Security to implement the morbidity-oriented risk structure compensation scheme in the statutory health insurance system. The Federal Office of Social Security also transmits data to the DIMDI for further use in the Information System for Health Care Data. Eligible institutions can apply for permission to analyze parts of the dataset, and after thorough scrutiny receive anonymized aggregate data [22]. 

A formal application for data usage and a data analysis script were submitted on 29 February 2016. At the time of the application, the most recent data available with information on the district level was from 2010. After adaption and a narrowing of our data request (e.g., removal of our initial request for high-resolution stratification by age), our application was thoroughly screened by DIMDI. We received the final dataset on 19 December 2018. 

We received strongly aggregated data of individuals with at least one prescription for metamizole, and the number of packs (of any size) prescribed. Data was also provided on age (five age brackets), sex, state (Bundesland), and on the number of persons in each stratum. For adults (18+), we also received the number of individuals with at least one prescription for metamizole and the number of packs prescribed on the district (Landkreis) level. Data was adjusted for the state of Mecklenburg-Western Pomerania to take into account a recent change in district boundaries [23]. It now corresponds to the current 402 districts in Germany. 

In descriptive analyses, the proportion of individuals with at least one prescription was calculated, as well as the mean number of prescribed packs. Stratification by age, sex, state and district were evaluated and presented using percentages, ranges, and interquartile ranges (IQR). 

All analyses and cartographic presentations were performed using SAS for Windows Version 9.4 (SAS Institute Inc., Cary, NC, USA).

## 3. Results

Of 68.4 million insured individuals (mean age 43.6 years, 53.0% female), a total of 5.5 million (8.1%) received at least one prescription for metamizole in 2010. A total of 12.2 million packs were prescribed, which translates to 0.2 packs per year per person, and 2.2 packs per metamizole user. Among individuals aged 17 years and younger, 1.5% received at least one prescription, whereas this proportion increased to 17.3% of those aged 70 years and older (Table 1). Women received metamizole more often than men (9.6 vs. 6.3%) with differences being most pronounced in the oldest age group (20.0 vs. 13.0%). 

The number of packs prescribed also increased with age, ranging from 1.2 in those under 18 years to 3.2 packs in the oldest age bracket. Female users were prescribed a higher mean number of packs (2.3 vs. 2.0), although, again, this difference resulted primarily from marked differences in persons aged 70 years and older.

The following regional analyses are confined to the 57.0 million adults (mean age: 50.2 years, 53.8% female). In this group, total prescription prevalence was 9.4% (women: 11.0%, men: 7.4%). Between states, values ranged from 7.0% in Saxony to 11.1% in Schleswig-Holstein (Table 2). 

**Figure 2:**
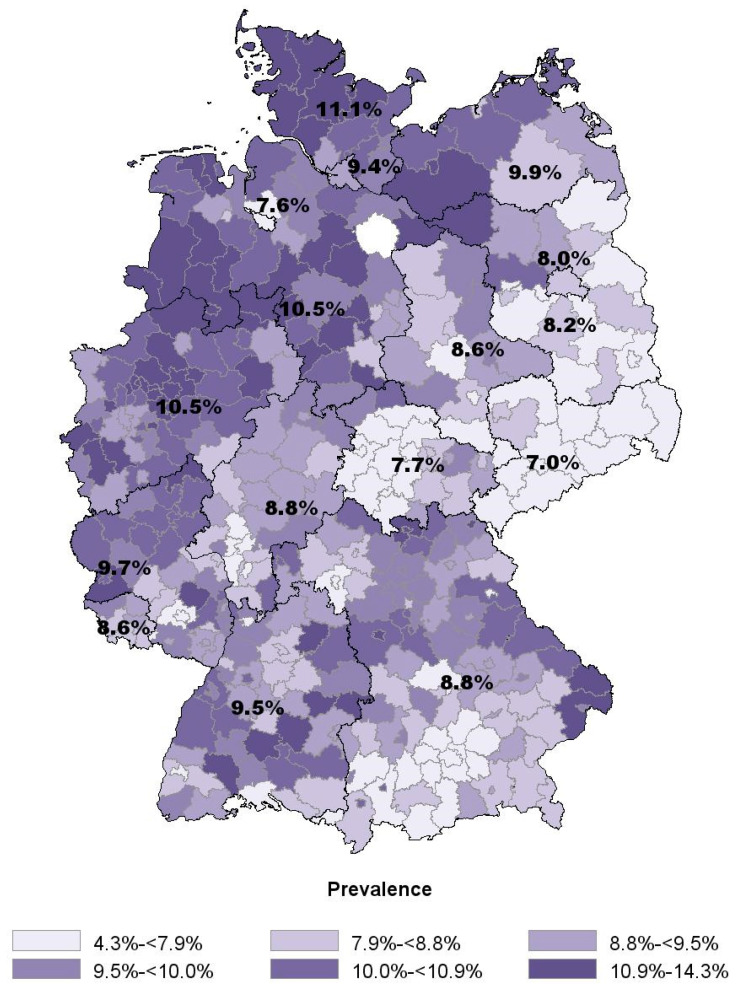
Proportion of adults (18+ years) with at least on prescription of metamizole in 2010 by district.

With the exception of Mecklenburg-Western Pomerania, all states of former East Germany had values below the national average. Apart from Bremen (7.6% in the city of Bremen, 9.6% in Bremerhaven), the highest prescription prevalence was recorded in the northwestern states. At the district level (Figure 2), values ranged from 4.3% (Suhl in Thuringia, mean age: 54.4 years, 53.8% female) to 14.3% (Uelzen in Lower Saxony, mean age: 51.9 years, 55.0% female). Within certain states, a marked difference between individual districts could be demonstrated, for example in Brandenburg and Bavaria.

## 4. Discussion

In our sample, one out of twelve members of statutory health insurance funds in Germany received at least one prescription for metamizole in 2010. Among adults, this proportion was 9.4%. Marked regional differences could be shown, with the lowest values mainly in the states of former East Germany. Northern states tended to have the highest prescription prevalences. Overall, our data confirms the relatively high relevance of metamizole in the outpatient setting described in the literature. For instance, a study with data from one health insurance fund from 2009 found metamizole prescribed to 6.8% of insurance members [15]. The value of 8.1% in our sample is likely to diverge from this due to the different year of reference, the limitation of previous data to one insurance fund, and the differences in the sex and age distribution: in our dataset, the mean age was four years higher and contained more females than the study cited above. Differences in prescription patterns by age and sex are comparable in both studies. Higher prescription prevalences with advancing age, as expected with an age-related increase in the occurrence of pain, were also found in another study [24]. In the context of nursing homes, metamizole plays an even greater role, although the available data is virtually limited to the German context. Some 40% of nursing home residents were found to receive metamizole [16,25]. It is the most widely used analgesic in this setting [16,25,26,27]. Again, sex and age differences comparable to our results were found in studies assessing nursing home residents [16,26].

Internationally, Germany plays a special role in the high market relevance of metamizole [24,25,28,29]. This is partially due to the ban of metamizole preparations in some countries [5,25]. However, even in countries where it is still available, metamizole seems to be used less than in Germany. In Croatia and Serbia in 2015, 1.3 and 4.0 500 mg tablets per inhabitant were distributed, respectively. In both countries, a decrease in usage was recorded between 2010 and 2015 [29]. In Switzerland, metamizole also plays a minor role compared to other analgesics, but similar to Germany, prescription volume has increased markedly over the last decade [24]. The average usage in Switzerland was 10.0 tablets of 500 mg tablets per inhabitant in 2013, compared to 13.4 tablets in Germany (see Figure 1). A study from Poland reported 75.5 million tablets of 500 mg in a one-year period (04/2006–03/2007) for an adult population of about 30 million [30]. This would translate to 2.5 tablets per adult and year, quite a lower value than the value in Germany for the same year (6.3 tablets). However, it is unclear whether other drug formulations or hospital usage were included in the numbers provided. Studies from neighboring countries where metamizole is a licensed drug (e.g., the Netherlands or Austria) have not been performed to the best of our knowledge.

Regional differences in our study were considerable, and were similar to previous studies on the state level [15]. For the first time, we were able to analyze data at the district level. Overall, eastern states were found to have the lowest proportion of inhabitants receiving metamizole prescriptions. Particularly in Saxony and Thuringia, nearly all districts had prevalences below the national average. This is remarkable given that in 1990, metamizole usage was much higher in the former German Democratic Republic compared to West Germany [31]. A contrary development seems to have occurred in the two parts of the country following reunification. However, given that, e.g., higher healthcare expenditures for specific drugs or higher proportions of vaccinated persons were found in the eastern compared to the western states [32,33], a general west–east divide with respect to drug prescriptions or healthcare utilization cannot be determined. In southern states, metamizole prescriptions were shown to be more frequent than in the east, but less frequent than in Northern Germany. The one exception here is the state of Bremen, where a critical stance towards metamizole has been traditionally held [34]. In some cases, large differences and particular patterns were observed within states. This is especially true for Bavaria where lower prescription prevalences were found in the southern districts on the Austrian border. Interestingly, remarkable regional differences exist in Switzerland as well, with metamizole prescribed most frequently in German-speaking cantons, and those bordering Germany [24]. On the other hand, the legal status in bordering countries does not seem to affect prescriptions in Germany. For example, metamizole is not sold in both France and Denmark [5], but is frequently prescribed in German districts on the French and Danish border. Further investigations are called for to shed light on the reasons for regional divergence in prescriptions.

Overall, we found that given the narrow spectrum of approved indications, metamizole prescribing in Germany was surprisingly high, especially in an international comparison. Furthermore, we were surprised by the dramatic increase in prescription numbers since 1991 which, for example, was much more pronounced than the increase observed for opioids. Additionally, the large regional differences found for metamizole prescriptions were remarkable. These cannot be explained by differences in physician density [35], or by a variation in physiotherapy spending per insurance member [36]. Since 1987, approved indications for the use of metamizole were limited to acute strong pain following injury or surgical intervention, colic, cancer pain, strong acute or chronic pain due to other causes if other therapeutic measures are not indicated or high fever failing to respond to other medications [13]. It seems unlikely that the number of patients with these indications has increased sufficiently over the last few years to explain the dramatic increase in metamizole prescriptions. It appears equally implausible that regional differences in prescriptions are grounded in differences in the distribution of patients with indications for usage. It is much more likely that metamizole is frequently employed in situations that fall outside its approved indications. However, to our knowledge, no study to date has evaluated this yet. Future studies should collect data on possible reasons for the widespread and growing metamizole use. Sufficient data on individual patient characteristics such as demographics, (contra) indications and comedication could enable an informed debate on whether satisfactory therapeutic alternatives exist, and what these alternatives could be. Previous studies have demonstrated that around 80% of prescriptions are issued by general practitioners (GPs), with proportions increasing further with advancing patient age [15]. Thus, future studies should focus primarily on GPs and the primary care setting.

The primary strength of this study is that for the first time, it provides analyses on the regional and individual level of prescription patterns of metamizole in Germany on the basis of a complete dataset of members of all statutory health insurance funds (over 85% of the German population, data on privately insured persons were not available). This is relevant, since individual funds differ in their geographic membership distribution and the sociodemographic and health-related characteristics of the insured population [37,38,39,40]. Results from analyses of individual funds can thus not be generalized to the German population at large. 

A main weakness of this study is the dataset pertaining to the year 2010. This time lag is caused by the complex and lengthy legal requirements and bureaucratic process for obtaining the dataset. Data are only available some four years after collection, and from submission to the delivery of the dataset, another 2.5 years came to pass. This delay is particularly important for our project, since in the meantime, there has been another marked increase in metamizole prescription volume: between 2010 and 2018, it increased from 123 million to 225 million DDD [19]. The current number of persons receiving metamizole is thus likely to be even higher than the one found in our sample. This data, however, is based on the annual Drug Prescription Report and does not provide information on the number of users, their respective age groups or on regional differences.

Based on our study data, it is not possible to infer whether prescribed metamizole was in fact taken. For example, in nursing homes metamizole is frequently prescribed *pro re nata* rather than for continuous use [16]. Information on whether patients live in nursing homes, as well as the medical specialty of prescribers were not available in the present data. A further limitation is the fact that due to a lack of access to the original dataset, no further analyses beyond those outlined in the initially submitted scripts were possible. This for example precluded us from performing a regional analysis of prescription patterns in those aged 65 and above, or from using a higher resolution of age brackets as a basis of analysis. Therefore, we did not include an age- or sex-stratified analysis at the district level in our manuscript. However, we performed an age- and sex-adjusted analysis at the state level and found comparable results: differences for adults ranged between 6.7% in Saxony and 11.1% in Schleswig-Holstein. Similarly, amongst those aged 70 and above, the lowest prescription prevalences were shown in Saxony (11.7%) and the highest values in Schleswig-Holstein (20.6%) (Supplemental Figure S1). Overall, regional differences in prescription practice could therefore not be explained by differences in age- or sex-distribution between states or districts. Lastly, patient level data on indications or contraindications regarding analgesic alternatives, on possible drug interactions with other medications or on adverse events were not available. Therefore, we are not able to evaluate whether metamizole was used appropriately in individual patient cases.

## 5. Conclusions

In 2010, about one out of twelve members of statutory health insurance funds in Germany received at least one prescription for metamizole. With a considerable increase in total prescription numbers over the last decade, it is likely that the current proportion of users is even higher. The high level of usage is surprising, especially given the narrow spectrum of approved indications for the use of metamizole. It seems probable that a large proportion of prescriptions in outpatient care are written off-label. We found remarkable regional differences that cannot be explained by factors on the patient side. 

## Figures and Tables

**Table 1:** Proportion of insured individuals with at least one prescription of metamizole, and the mean number of packs per user in 2010, by age and sex.

Age Bracket (Number of Persons)	Prevalence of Use			Mean Number of Packs Per User		
	Overall	Male	Female	Overall	Male	Female
<18 years (n = 11,067,762)	1.5%	1.2%	1.7%	1.2	1.2	1.2
18–29 years (n = 9,887,789)	5.3%	4.2%	6.5%	1.2	1.2	1.2
30–49 years (n = 19,184,926)	6.7%	5.8%	7.5%	1.5	1.5	1.5
50–69 years (n = 17,154,890)	9.5%	8.1%	10.7%	2.0	2.1	2.0
70+ years (n = 11,132,097)	17.3%	13.0%	20.0%	3.2	2.8	3.4
Overall (n = 68,427,464)	8.1%	6.3%	9.6%	2.2	2.0	2.3

**Table 2:** Proportion of adults (18+ years) with at least one prescription of metamizole, and mean number of packs per user by state and distribution by district in 2010.

Federal State (Population; Number of Districts)	Mean Age		Proportion of Females		Prevalence of Use			Mean Number of Packs Per User		
	Overall	Range **	Overall	Range ^§^	Overall	Range ^†^	Median (IQR)	Overall	Range ^‡^	Median (IQR)
Saxony (n = 3,238,095; 13 districts)	52.6	49.9–54.2	53.5%	52.6–54.2%	7.0%	6.1–8.8%	6.8% (6.2–7.5)	1.9	1.8–2.0	1.9 (1.9–1.9)
Bremen (n = 475,025; 2 districts)	50.0	49.9–50.6	53.8%	52.9–53.9%	7.6%	7.1–9.6%	–	2.5	2.5–2.6	–
Thuringia (n = 1,713,973; 23 districts)	52.3	49.3–54.4	52.9%	51.3–55.1%	7.7%	4.3–10.1%	7.8% (6.9–9.1)	2.0	1.8–2.3	2.0 (1.9–2.2)
Berlin (n = 2,369,412; 1 district)	48.9	–	54.1%	–	8.0%	–	–	2.2	–	–
Brandenburg (n = 1,883,024; 18)	52.1	49.4–53.5	53.1%	52.1–54.7%	8.2%	6.5–11.0%	8.0% (7.5–8.7)	2.1	2.0–2.4	2.1 (2.1–2.2)
Saarland (n = 726,682; 6 districts)	51.2	50.8–51.6	53.8%	53.5–54.3%	8.6%	8.1–9.9%	8.6% (8.2–9.3)	2.0	1.9–2.1	2.0 (2.0–2.1)
Saxony–Anhalt (n = 1,814,462; 14 districts)	52.7	51.4–54.4	53.1%	52.3–54.4%	8.6%	7.5–9.7%	8.7% (8.0–9.6)	2.0	1.8–2.3	2.0 (1.9–2.0)
Hesse (n = 4,160,092; 26 districts)	49.9	47.4–52.4	53.9%	52.1–55.7%	8.8%	7.6–10.9%	9.1% (8.2–9.4)	2.2	1.9–2.5	2.2 (2.1–2.2)
Bavaria (n = 8,360,769; 96 districts)	49.3	46.7–52.9	54.4%	52.3–60.4%	8.8%	6.2–12.6%	9.2% (8.3–9.9)	2.2	1.8–2.5	2.2 (2.1–2.3)
Hamburg (n = 1,206,785; 1 district)	48.3	–	54.5%	–	9.4%	–	–	2.5	–	–
Baden–Württemberg (n = 7,161,296; 44 districts)	49.6	46.8–52.9	54.0%	52.5–57.2%	9.5%	7.6–12.8%	9.5% (8.8–10.2)	2.3	2.0–2.7	2.3 (2.2–2.4)
Rhineland–Palatinate (n = 2,713,894; 36 districts)	50.3	47.5–52.4	54.0%	52.6–56.0%	9.7%	5.8–11.2%	9.7% (9.0–10.5)	2.2	1.9–2.5	2.2 (2.1–2.3)
Mecklenburg–Western Pomerania (n = 1,242,670; 8 districts)	51.8	48.9–53.1	53.1%	52.3–54.7%	9.9%	7.9–13.3%	10.1% (8.7–10.7)	2.2	2.0–2.7	2.1 (2.0–2.5)
Lower Saxony (n = 5,446,299; 46 districts)	50.2	46.8–53.6	53.7%	51.9–55.0%	10.5%	6.9–14.3%	10.5% (9.8–11.4)	2.3	2.1–2.8	2.3 (2.3–2.4)
North Rhine–Westphalia (n = 12,523,815; 53 districts)	50.0	47.0–52.0	53.7%	52.0–55.7%	10.5%	8.2–12.7%	10.5% (9.7–11.3)	2.3	2.1–2.6	2.3 (2.2–2.4)
Schleswig-Holstein (n = 1,939,155; 15 districts)	50.5	47.4–52.3	54.6%	53.6–55.7%	11.1%	9.4–13.5%	11.4% (10.2–12.6)	2.5	2.3–2.9	2.5 (2.4–2.6)
Germany (n = 56,975,448; 402 districts) *	50.2	46.7–54.4	53.8%	51.3–60.4%	9.4%	4.3–14.3%	9.5% (8.4–10.4)	2.2	1.8–2.9	2.2 (2.1–2.3)

* For about 0.7% of adults no valid information on federal state and district were available. These persons were excluded from the analysis. ** The range displays the districts’ lowest and highest mean age within the respective state. ^§^ The range displays the districts’ lowest and highest proportion of females within the respective state. **^†^** The range displays the districts’ lowest and highest prevalence of use within the respective state. **^‡^** The range displays the districts’ lowest and highest mean number of packages per user within the respective state.

## Supplementary Materials

The following are available online at https://www.mdpi.com/1660-4601/17/11/3892/s1, Figure S1: Proportion of adults with at least on prescription of metamizole in 2010 by state and age.
