# Supplementary material for: Regional Differences in Prescribing Patterns of Metamizole in Germany Based on Data from 70 Million Persons
Source: Int J Environ Res Public Health. 2020 May 30;17(11):3892. doi: 10.3390/ijerph17113892 (PMC7312502; doi:10.3390/ijerph17113892)
Supplement: Supplementary file 1 [file ijerph-17-03892-s001.pdf]

Regional differences in prescribing patterns of metamizole in Germany based on data from 70 million persons

Falk Hoffmann, Carsten Bantel, Frederik Tilmann von Rosen and Kathrin Jobski

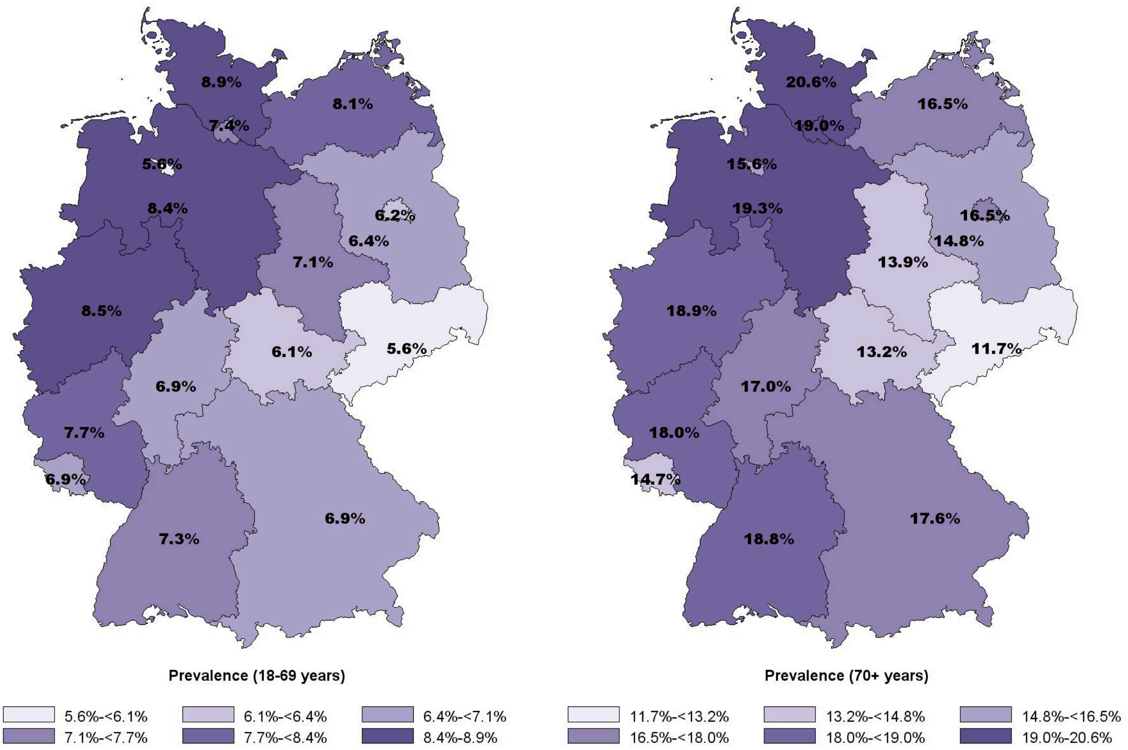

Supplemental Figure S1. Proportion of adults with at least on prescription of metamizole in 2010 by state and age
